# Ponatinib enhances anticancer drug sensitivity in MRP7-overexpressing cells

**DOI:** 10.3892/or.2014.3002

**Published:** 2014-01-28

**Authors:** YUE-LI SUN, PRIYANK KUMAR, KAMLESH SODANI, ATISH PATEL, YIHANG PAN, MARIA R. BAER, ZHE-SHENG CHEN, WEN-QI JIANG

**Affiliations:** 1 State Key Laboratory of Oncology in South China, Guangzhou, Guangdong, P.R. China; 2 Department of Medical Oncology, Sun Yat-sen University Cancer Center, Guangzhou, Guangdong, P.R. China; 3 Department of Pharmaceutical Sciences, College of Pharmacy and Health Sciences, St. John’s University, Queens, New York, NY; 4 Cytogenetics Laboratory, Department of Pathology and Laboratory Medicine, Weill Cornell Medical College, New York, NY; 5 University of Maryland Greenebaum Cancer Center and Department of Medicine, University of Maryland School of Medicine, Baltimore, MD, USA

**Keywords:** multidrug resistance, MRP7, ponatinib, TKI, MDR, ABC transporter

## Abstract

The presence of acquired multidrug resistance (MDR) is one of the primary impediments to the success of chemotherapy. MDR is often a result of overexpression of ATP-binding cassette (ABC) transporters, which are involved in the extrusion of therapeutic drugs. Recently, it was shown that several ABC transporters could be modulated by specific tyrosine-kinase inhibitors (TKIs). Ponatinib, a multi-targeted TKI, inhibits the activity of BCR-ABL with very high potency and broad specificity, including the T315I mutation which confers resistance to other TKIs. It was reported that ponatinib was capable of reversing breast cancer resistance protein (BCRP)- and P-glycoprotein (P-gp)-mediated MDR. In the present study, we report for the first time that ponatinib also potentiates the cytotoxicity of widely used therapeutic substrates of MRP7, such as paclitaxel, docetaxel, vincristine and vinblastine. Ponatinib significantly enhances the accumulation of [^3^H]-paclitaxel in cells expressing MRP7. Furthermore, accumulation of [^3^H]-paclitaxel was achieved by inhibition of MRP7-mediated transport. Ponatinb limited drug export via MRP7 by multiple mechanisms. In addition to inhibition of pump function, ponatinib also downregulated MRP7 protein expression in a time- and concentration-dependent manner. Thus, ponatinib may represent a potential reversal agent for the treatment of MDR and may be useful for combination therapy in MDR cancer patients in clinical practice.

## Introduction

**Figure 1:**
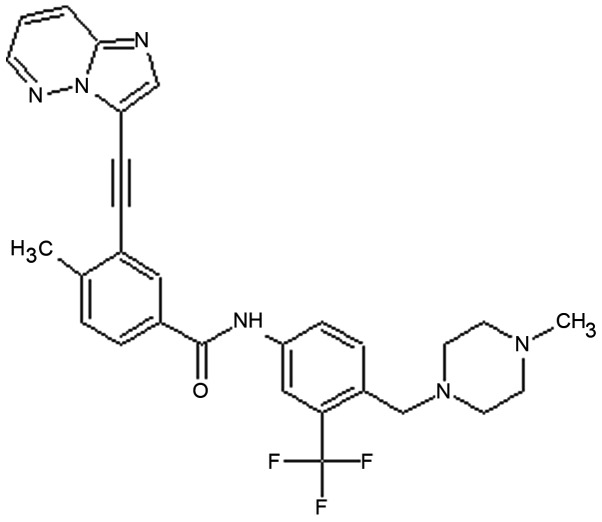
Chemical structure of ponatinib.

The use of imatinib in the treatment of patients with chronic myeloid leukemia (CML) must be ranked as one of the great medical success stories of the past 30 years (1). However, ~20% of patients with CML do not respond to treatment with imatinib either initially or as a result of acquired resistance (2). Dasatinib and nilotinib are treatment options for CML patients for whom treatment with imatinib has failed owing to resistance and/or intolerance. However, 22–33% of those patients had to discontinue by two years due to adverse events, treatment failure or other causes (3). Resistance to tyrosine kinase inhibitors (TKIs) in patients with CML and Philadelphia chromosome-positive acute lymphoblastic leukemia (Ph-positive ALL) is frequently caused by mutations in the BCR-ABL kinase domain (4). Ponatinib (the chemical structure is shown in Fig. 1) was designed by ARIAD using a computational and structure-based drug design platform to inhibit the activity of BCR-ABL with very high potency and broad specificity. Ponatinib targets not only native BCR-ABL but also its isoforms that carry mutations which confer resistance to treatment with existing TKIs, including the T315I which causes uniform resistance to TKIs (5). The Food and Drug Administration (FDA) approved ponatinib to treat two forms of drug-resistant leukemia, CML and Ph-positive ALL, in December 2012.

The ATP-Binding Cassette (ABC) transporter superfamily is one of the largest and most highly conserved protein families, with structural features and mechanisms of action conserved from prokaryotes to humans (6). ABC transporters are known to account for the multidrug resistant (MDR) phenotype of various cancer cells. They are capable of recognizing and extruding a broad range of compounds, without relation to chemical structure or cellular target (7). When overexpressed in cancer cells, ABC transporters reduce intracellular drug concentrations below the effective cytotoxic threshold and induce drug resistance (8). The major ABC proteins that are widely accepted to be responsible for the MDR phenotype of cancer cells are P-glycoprotien (P-gp, also called MDR1 or ABCB1), multidrug resistance proteins (MRPs, also called ABCCs) and breast cancer resistance protein (BCRP, also called ABCG2, BCRP, MXR or ABCP) with each having promiscuous and overlapping substrate recognition spectra (9).

ABC transporters have recently been recognized as important determinants of the general ADME-Tox (absorption, distribution, metabolism, excretion and toxicity) properties of small molecule TKIs, as well as key factors in resistance against targeted anticancer therapeutics (10). The interaction of numerous clinically applied TKIs with various ABC transporters is complicated. TKIs may be extruded out of the cell by ABC transporters. However, TKIs may also directly inhibit ABC transporters, thereby inducing sensitization to chemotherapy agents (11,12). In some cases, TKI compounds may behave both as substrates and inhibitors of a given transporter, depending on the concentration range applied (13). Imatinib was reported to be able to inhibit the transport of substrates of P-gp, BCRP, MRP1 and MRP7 (14–16). Nilotinib was shown to be an inhibitor of P-gp, BCRP and MRP7 both *in vitro* and *in vivo* (16–18). Similarly, lapatinib, which is used for the treatment of Her-2 positive advanced or metastatic breast cancer, is an inhibitor of P-gp, BCRP and MRP7 (19,20). The third-generation TKI ponatinib also enhances uptake of substrates of BCRP and P-gp, but not MRP1, with a greater effect on BCRP than on P-gp (21).

MRP7 (also called ABCC10) belongs to the C subfamily of ABC transporters. It confers *in vitro* resistance to a wide range of clinically used anticancer drugs, including taxanes, vinca alkaloids, nucleoside analogs and epothilone B (22). *In vivo*, absence of this transporter also sensitized animals to taxanes in an Abcc10^−/−^ mouse model, indicating that MRP7 may function as a major determinant of taxane sensitivity (23). These findings suggest that modulation of MRP7 may have clinical value in management of human cancers which are treated with taxane-contained regimens. Whether ponatinib has potential efficacy in reversing MRP7-mediated MDR was explored in the present study. We found that ponatinib was able to reverse MRP7-mediated MDR at a low concentration by both blocking MRP7 function and downregulating its expression.

## Materials and methods

### Reagents

Paclitaxel, docetaxel, vincristine, vinblastine, dimethyl sulfoxide (DMSO) and 1-(4,5-dimethylthiazol-2-yl)-3,5-diphenylformazan (MTT) were purchased from Sigma-Aldrich. Cepharanthine was kindly provided by the Kakenshoyaku Co. [^3^H]-paclitaxel (38.9 Ci/mmol) was obtained from Moravek Biochemicals. Ponatinib was acquired from Selleck Chemicals.

### Cell lines and cell culture

MRP7 expression vector (HEK/MRP7) and parental plasmid (HEK/pcDNA) were transfected into human embryonic kidney 293 (HEK293) cells by electroporation as we previously reported (24). Transfected cells were selected in DMEM containing 2 mg/ml G418. MRP7 protein was detected by immunoblot analysis. All cell lines were grown as adherent monolayers in DMEM supplemented with 10% fetal bovine serum (FBS), 200 U/ml penicillin and 200 U/ml streptomycin (HyClone). All cell lines were grown at 37°C in 5% CO_2_ under humidifying conditions.

### MTT assay

The sensitivity of cells to anticancer drugs was measured by an MTT colorimetric assay with minor modifications from that previously described (25). Cells were harvested with trypsin and resuspended at a final concentration of 6×10^3^ cells/well. After incubation in DMEM supplemented with 10% FBS at 37°C for 24 h, ponatinib (0.1, 0.25 or 0.5 μM, 20 μl/well) or the MRP7 inhibitor cepharanthine (26) (2.5 μM, 20 μl/well) were added 1 h prior to the addition of anticancer drugs at different concentrations (20 μl/well). After 68 h of incubation, 20 μl of MTT solution (4 mg/ml) was added to each well, and the plate was incubated for another 4 h, allowing viable cells to convert the yellow-colored MTT into dark blue formazan crystals. Then the medium was aspirated, and 100 μl DMSO was added to each well to dissolve the formazan crystals. The absorbance was determined at 570 nm and 630 nm by an Opsys microplate reader (Dynex Technologies). The degree of resistance was calculated by dividing the IC_50_ (concentrations required to inhibit growth by 50%) for the MDR cells by that of the parental sensitive cells. The IC_50_ values were calculated from the survival curves using the Bliss method (27).

### [^3^H]-paclitaxel accumulation and efflux

Intracellular paclitaxel accumulation and efflux were measured in HEK/pcDNA cells and HEK/MRP7 cells. For the accumulation assay, the cells were trypsinized and three aliquots (5×10^6^ cells) from each cell line were resuspended in medium. To measure drug accumulation, cells were preincubated in DMEM in the presence or absence of ponatinib (at 0.5 μM) or cepharanthine (at 2.5 μM) for 2 h, washed and then incubated with 0.01 μM [^3^H]-paclitaxel with or without reversing agents for another 2 h at 37°C. The cells were pelleted at 4°C, washed twice with 10 ml ice-cold PBS, and lysed in 10 mM lysis buffer (pH 7.4, containing 1% Triton X-100 and 0.2% SDS). Radioactivity was measured in a liquid scintillation counter. For the efflux study, cells were incubated with 0.01 μM [^3^H]-paclitaxel according to the method for the accumulation study. After being washed twice with cold PBS, the cells were cultured in fresh DMEM with or without 0.5 μM ponatinib at 37°C. After 0, 30, 60 or 120 min, aliquots of cells were removed and immediately washed with ice-cold PBS. The cell pellets were collected for radioactivity measurement in a Packard TRI-CARB^®^ 1900CA liquid scintillation analyzer (Packard Instrument Co.).

### Western immunoblot analysis

To determine the effect of ponatinib on the expression of MRP7, HEK/MRP7 cells were incubated with 0.5 μM ponatinib for 0, 2, 4, 6, 8, 12 and 24 h or 0, 0.1, 0.25 and 0.5 μM for 24 h. After treatment, the cells were harvested and rinsed three times with ice-old PBS. Cell extracts were prepared by incubating cells for 30 min on ice with radioimmunoprecipitation assay (RIPA) buffer (PBS with 0.1% SDS, 1% Nonidet P-40, 0.5% sodium deoxycholate and 100 mg/ml p-aminophenylmethylsulfonyl fluoride) with occasional rocking, followed by centrifugation (12,000 g, 4°C for 20 min). The supernatant containing total cell lysates was stored at −80°C prior to experiments. Cell lysates containing identical amounts of total protein (25 μg for different time treatment group and 60 μg for different concentration treatment group) were resolved by sodium dodecyl sulfate polycrylamide gel electrophoresis (SDS-PAGE) and transferred onto polyvinylidene fluoride (PVDF) membranes. After incubation in a blocking solution containing 5% non-fat milk in TBST buffer [10 mmol/l Tris-HCl (pH 8.0), 150 mmol/l NaCl, and 0.1% Tween-20] for 2 h at room temperature, the membranes were immunoblotted overnight with primary antibodies against MRP7 (1:200 dilution; Santa Cruz Biotechnology) or GAPDH (1:1,000 dilution; Cell Signaling Technology) at 4°C, and then the membranes were washed five times for 5 min/each time with TBST buffer and incubated at room temperature with horseradish peroxidase (HRP)-conjugated secondary antibody (1:1,000 dilution) for 2 h. The protein-antibody complex was detected by chemiluminescence. Densitometry analysis was performed using the Quantity One software (Bio-Rad) and MRP7 immunoblots were normalized to GAPDH immunoblots for each sample analyzed.

### Reverse transcription polymerase chain reaction (RT-PCR)

Total RNA was extracted from HEK/pcDNA and HEK/MRP7 cells using TRIzol according to the manufacturer’s instructions. Reverse transcription was performed with 2 μg total RNA in a volume of 20 μl by Transcription System (Promega) according to the manufacturer’s instructions. The sequences of the MRP7 and GAPDH primers were as follows: MRP7 (303 bp) sense: 5′-GGCTCCGGCAAGTCTTCCCTGTT-3′ and antisense: 5′-AGATAAGCTCCGGCCCCCCTCACC-3′. GAPDH (322 bp) sense: 5′-CGGGAAGCTTGTCATCAA TGG-3′ and antisense 5′-GGCAGTGATGGCATGGACTG-3′. PCR was carried out with GoTaq^®^ Master Mixes and the reaction conditions were 94°C for 30 sec, 60°C for 40 sec (GAPDH) or 65°C for 40 sec (MRP7), 72°C for 45 sec with 35 cycles. The PCR products were separated by agarose gel electrophoresis. The gel was stained with 0.5 μg/ml ethidium bromide and the bands were visualized under UV light.

### Statistical analysis

All experiments were repeated at least three times. Statistical differences were determined by the two-tailed Student’s t-test, and were deemed significant if P-value was <0.05.

## Results

### Ponatinib significantly enhanced the drug sensitivity of MRP7-overexpressing cells

**Figure 2:**
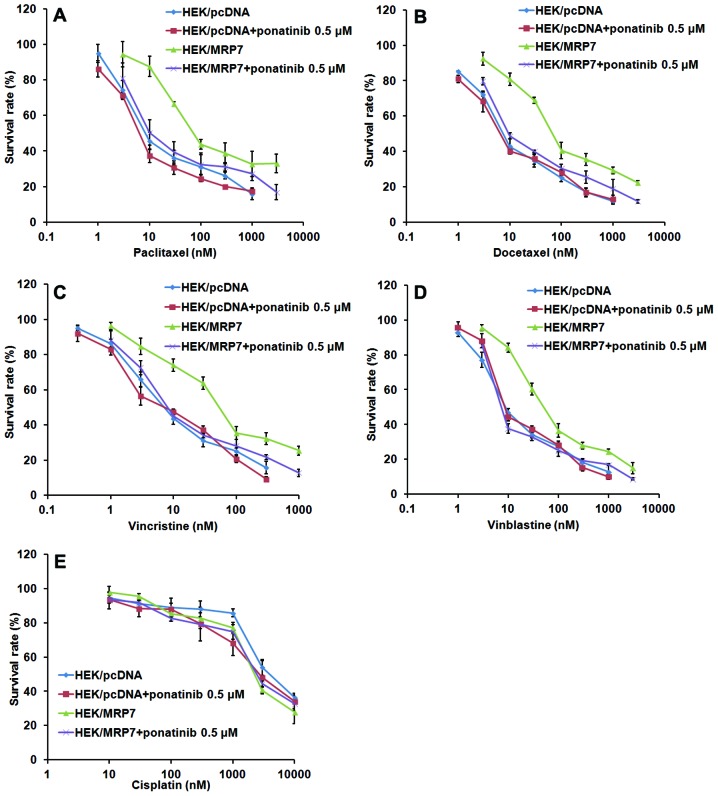
Effect of ponatinib on sensitivity of HEK/pcDNA and HEK/MRP7 cells to anticancer drugs. HEK/pcDNA cells and HEK/MRP7 cells were cultured for 24 h before ponatinib was added. After 1-h incubation, (A) paclitaxel, (B) docetaxel, (C) vincristine, (D) vinblastine or (E) cisplatin was added and the cultures were incubated for 72 h. Cell viability was measured by the MTT assay. Results are representative of 3 independent experiments, respectively.

In order to determine if ponatinib could reverse ABC transporter-mediated MDR, we treated both the parental cell line HEK/pcDNA and the resistant cell lines HEK/MRP7 with ponatinib 1 h prior to anticancer drugs, then measured the IC_50_ of anticancer drugs in parental cells and resistant cells using the MTT assay. Compared to parental HEK/pcDNA cells, HEK/MRP7 cells exhibited significant resistance to various MRP7 substrates, including paclitaxel (9.14-fold), docetaxel (8.75-fold), vincristine (5.65-fold) and vinblastine (5.99-fold). As shown in Table I, ponatinib at 0.1, 0.25 and 0.5 μM produced a concentration-dependent increase in sensitivity to paclitaxel, docetaxel, vincristine and vinblastine in MRP7-overexpressing cells. However, ponatinib did not significantly alter the cytotoxity of the tested drugs in the parental sensitive HEK/pcDNA cells. In addition, ponatinib did not significantly alter the IC_50_ value of cisplatin, which is not a substrate of MRP7. Curves clearly shifted significantly to the left side after coincubation of HEK/MRP7 cells with ponatinib at 0.5 μM (Fig. 2).

### Ponatinib significantly increases the accumulation of intracellular [^3^H]-paclitaxel in MRP7-overexpressing cells

**Figure 3:**
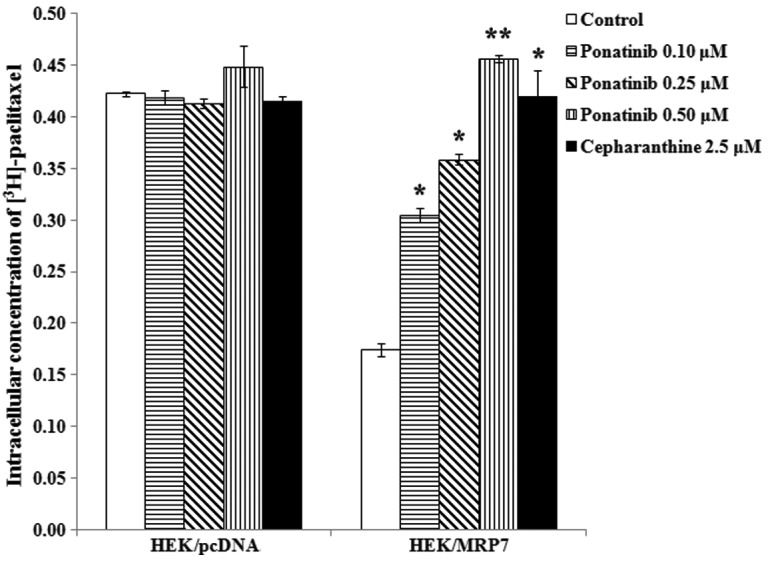
Effect of ponatinib on accumulation of [^3^H]-paclitaxel in HEK/pcDNA and HEK/MRP7 cells. Accumulation of [^3^H]-paclitaxel was measured after preincubation in the presence or absence of ponatinib at 0.1, 0.25 and 0.5 μM or cepharanthine at 2.5 μM for 2 h at 37°C, followed by incubation with 0.1 μM [^3^H]-paclitaxel with or without the reversal agents for another 2 h at 37°C. The cells were then collected and the intracellular level of [^3^H]-paclitaxel was measured by scintillation counting. Experiments were performed in triplicate, and results are expressed as mean ± SD. ^*^P<0.05 vs. the respective untreated controls.

In order to determine the effect of ponatinib on the function of MRP7, we measured the accumulation of [^3^H]-paclitaxel with or without ponatinib in HEK/pcDNA and HEK/MRP7 cells. The intracellular concentration of [^3^H]-paclitaxel in HEK/MRP7 was ~41.2% of that in parental HEK/pcDNA cells. After the cells were incubated with ponatinib at 0.1, 0.25 and 0.5 μM and cepharanthine at 2.5 μM for 4 h, intracellular [^3^H]-paclitaxel accumulation was significantly increased in HEK/MRP7 by 1.75-, 2.1-, 2.6- and 2.4-fold, respectively. However, the intracellular level of [^3^H]-paclitaxel in HEK/pcDNA was not altered by either ponatinib or cepharanthine (Fig. 3).

### Ponatinib blocks the efflux of [^3^H]-paclitaxel in MRP7-overexpressing cells

**Figure 4:**
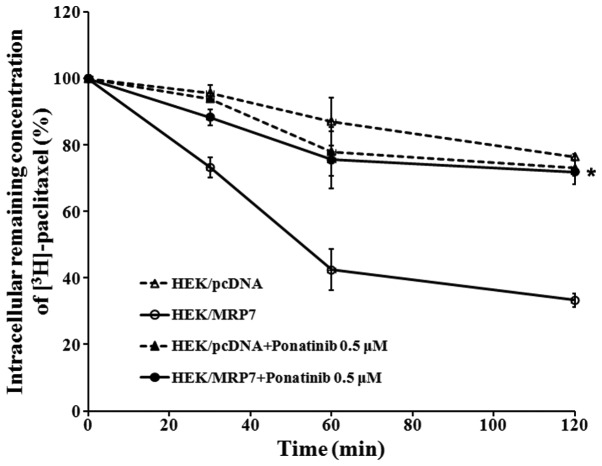
Effect of ponatinib on the efflux of [^3^H]-paclitaxel in HEK/pcDNA and HEK/MRP7 cells. The efflux assay was performed as described in Materials and methods. The values at 0 min were set as 100%. Each data point represents the mean ± SD of three independent experiments, each performed in triplicate. ^*^P<0.05, vs. HEK/MRP7 cells without ponatinib treatment.

To establish whether the increased intracellular [^3^H]-paclitaxel accumulation in MRP7-overexpressing cells caused by ponatinib was due to inhibition of [^3^H]-paclitaxel efflux, we performed a time course study to determine the remaining intracellular concentration of [^3^H]-paclitaxel in the presence of ponatinib. As expected, HEK/MRP7 cells released a significantly higher percentage of accumulated [^3^H]-paclitaxel compared to HEK/pcDNA cells. When ponatinib at 0.5 μM was added to HEK/MRP7 cells, it significantly blocked the intracellular [^3^H]-paclitaxel efflux at different time periods (0, 30, 60 and 120 min), but had no effct in the parental HEK/pcDNA cells. The accumulation of [^3^H]-paclitaxel at 0 min was set at 100% and at 30, 60 and 120 min, the percentages of the intracellular [^3^H]-paclitaxel that remained in HEK/MRP7 cells were 73.32, 42.51 and 33.28%, respectively, in the absence of ponatinib. When HEK/MRP7 cells were incubated with ponatinib, the percentages at 30, 60 and 120 min increased to 88.35, 75.54 and 71.89%, respectively (Fig. 4).

### Ponatinib downregulates MRP7 protein expression in a concentration- and time-dependent manner

**Figure 5:**
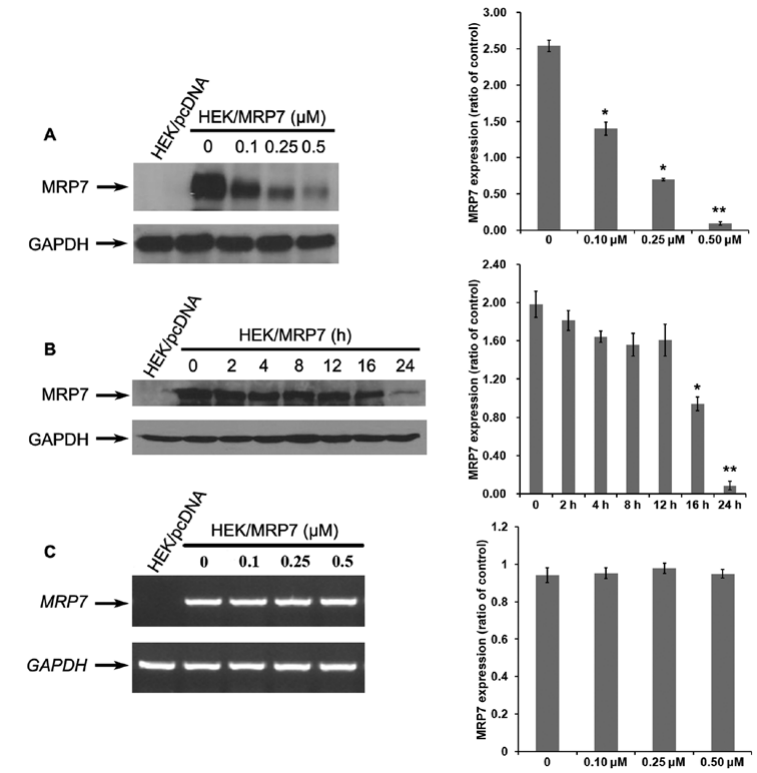
Effect of ponatinib on protein and mRNA expression of MRP7 in HEK/pcDNA and HEK/MRP7 cells. (A) The effect of ponatinib treatment for 24 h at different concentrations on the protein expression of MRP7 in HEK/pcDNA and HEK/MRP7 cells. (B) The effect of 0.5 μM ponatinib incubation on the protein expression of MRP7 in HEK/pcDNA and HEK/MRP7 cells at 0, 2, 4, 8, 12, 16 and 24 h. (C) mRNA levels of MRP7 in HEK/pcDNA and HEK/MRP7 cells treated with 0.1, 0.25 and 0.5 μM ponatinib for 24 h. GAPDH was used as a loading control. Protein or mRNA levels of MDR7 normalized to those of GAPDH are shown on the right. Results are presented as the mean ± SD. P-values were obtained using analysis of variance by comparing the relative amounts of protein or mRNA in cells treated with ponatinib with those in untreated control cells. Results representative of three independent experiments are shown. ^*^P<0.05; ^**^P<0.01.

MRP7-mediated MDR can be reversed by either inhibiting its transport function or decreasing the protein expression level of MRP7. To further ascertain the mechanism of reversal by ponatinib, we also treated MRP7-overexpressing cells with ponatinib at 0.5 μM for 0, 2, 4, 6, 8, 12 and 24 h or at 0, 0.1, 0.25 and 0.5 μM for 24 h to test the expression of MRP7. Treatment of HEK/MRP7 cells with ponatinib at 0, 0.1, 0.25 and 0.5 μM for 24 h led to downregulation of MRP7 expression in a concentration-dependent manner (Fig. 5A). The results shown in Fig. 5B indicated that ponatinib treatment for more than 16 h significantly downregulated MRP7 protein expression in a time-dependent manner.

Protein downregulation could occur either at the transcriptional or the post-transcriptional level. RT-PCR was conducted to ascertain whether the mRNA level of MRP7 was also downregulated in HEK/MRP7 cells treated with ponatinib at 0.5 μM. As shown in Fig. 5C, mRNA levels of MRP7 did not decrease significantly in the presence of ponatinib even after 24 h. These results indicated that ponatinib downregulated MRP7 expression at the post-transcriptional level.

## Discussion

Increased expression and functionality of ABC transporters are common features of cancer cells and often underlie chemoresistance (28–30). Thus, the persistence of cancer cells expressing abnormally high levels of ABC transporters is associated with dismal prognosis (31,32). ABC pumps constitute an attractive therapeutic target, as revealed by multiple preclinical and clinical studies showing that inhibitors of drug efflux sensitize cancer cells to chemotherapy (31,33). Nevertheless, the clinical use of ABC transporter inhibitors has been limited by unacceptable side-effects, drug interactions or concerns about long-term safety (34–36). The attractive feature of the novel BCR-ABL inhibitor ponatinib is that its toxicities were generally mild. The common side-effects were rashes, dry skin, abdominal pain, headache and constipation, all seen in ~40% of patients, and most cases were reported to be mild (37). Ponatinib has been found to increase the cytotoxicity of substrates of BCRP and P-gp in BCRP- and P-gp overexpressing cells (21).

In the present study, we found that ponatinib was a potent reversal agent for MRP7-mediated MDR. It could increase the cytotoxic response of MRP7-expressing cells to chemotherapeutic agents, including paclitaxel, docetaxel, vincristine and vinblastine, at a clinically achievable concentration. Consistent with the cytotoxicity result, drug accumulation studies also showed that ponatinib significantly increased the intracellular accumulation of [^3^H]-paclitaxel in MRP7-overexpressing cells in a concentration-dependent manner. Efflux data indicated that this increased intracellular accumulation of [^3^H]-paclitaxel in a short time period (4 h) was caused by direct inhibition of MRP7 transport function, as MRP7 protein downregulation caused by ponatinib only occurred after 16-h incubation. These findings indicate that the potent BCR-ABL inhibitor ponatinib could reverse MRP7-mediated MDR by inhibiting the function of MRP7 in a short time period and also by downregulating protein expression after longer incubation.

The molecular mechanisms underlying the protein regulation of MRP7 still need further study. Our RT-PCR data showed that *MRP7* mRNA level did not decrease in conjunction with protein downregulation, which indicates that protein regulation occurred at a post-transtriptional level. Protein degradation and translocation may be considered and confirmed by further studies. Niu *et al* (38) reported that low molecular weight heparin (LMWH) prevented chemoresistance of lung SP cells by reducing BCRP protein expression through the ubiquitin-proteasome pathway, as the proteasomal inhibitor MG132 restored ABCG2 protein expression, while the lysosomal inhibitors leupeptin and pepstatin A did not. In addition, it was also shown that significant enhancement of the intracellular accumulation of calcein was able to induce downregulation of P-gp (39). PI3k/Akt and NF-κB are the common pathways involved in the regulation of ABC transporter expression. PAPP-A decreased the expression of ABCA1 and ABCG1 in THP-1 macrophage-derived foam cells through the PI3K/Akt signaling pathway (40). Bortezomib reversed leukemia cell MDR in a concentration-dependent manner as the result of reduction of P-gp expression through the NF-κB pathway (41).

MRP7 expression is increased in many cancers in association with stage and prognosis. MRP7 expression level was upregulated in non-small cell lung cancer (NSCLC) compared to normal lung tissues, and the higher expression was correlated with advanced pathological grades and TNM stage in adenocarcinoma (42). Oguri *et al* (43) found that MRP7 can affect *in vivo* tissue sensitivity to taxanes, and could be used as a predictive marker of resistance to paclitaxel in NSCLC. A similar phenomenon was also observed in hepatocellular carcinoma; the MRP7 expression level was also elevated compared to normal adjacent healthy liver samples (44). These findings indicate that MRP7 expression might be a biomarker or regulator of treatment response in certain cancers and modulation of MRP7 expression and function may have clinical value in cancer treatment.

In conclusion, we showed here for the first time that ponatinib inhibits MRP7 function and downregulates MRP7 protein expression, hence facilitating the intracellular accumulation of selected chemotherapeutic agents and increasing their cytotoxicity. Clearly, additional studies of mechanism and animal study are needed, and it will be worthwhile to explore whether ponatinib can increase the cytotoxicity of anticancer therapeutic agents *in vivo*. The clinical application of ponatinib in MDR cancer patients may have great potential.

## Figures and Tables

**Table I:** Effect of ponatinib and cepharanthine on the cytotoxicity of paclitaxel, docetaxel, vincristine, vinblastine, and cisplatin in MRP7-transfected cells.

	IC_50_ ±SD^a^ (resistance fold)	
	
Compounds	HEK/pcDNA	HEK/MRP7
Paclitaxel (μM)	9.16±0.53 (1.00)	83.75±3.77 (9.14)
Ponatinib (0.10)	9.11±0.53 (0.99)	19.37±1.01 (2.11)^c^
Ponatinib (0.25)	8.87±0.60 (0.97)	10.02±1.57 (1.09)^c^
Ponatinib (0.50)	7.65±0.68 (0.84)	9.25±0.22 (1.01)^c^
Cepharanthine (2.5)	7.79±0.97 (0.85)	8.46±1.07 (0.92)^c^
Docetaxel (μM)	8.74±0.82 (1.00)^b^	76.44±5.53 (8.75)
Ponatinib (0.10)	7.78±1.05 (0.89)	16.30±1.02 (1.86)^c^
Ponatinib (0.25)	7.66±0.47 (0.88)	10.30±0.85 (1.18)^c^
Ponatinib (0.50)	8.26±1.04 (0.95)	7.16±0.67 (0.82)^c^
Cepharanthine (2.5)	8.12±0.88 (0.93)	7.50±1.35 (0.86)^c^
Vincristine (μM)	7.82±0.85 (1.00)^b^	44.19±3.73 (5.56)
Ponatinib (0.10)	8.84±0.79 (1.13)	21.46±2.36 (2.74)^c^
Ponatinib (0.25)	7.38±0.93 (0.94)	9.02±0.75 (1.15)^c^
Ponatinib (0.50)	8.12±0.44 (1.04)	7.87±0.82 (1.01)^c^
Cepharanthine (2.5)	7.21±0.42 (0.92)	8.28±0.79 (1.06)^c^
Vinblastine (μM)	9.49±0.69 (1.00)^b^	56.79±7.41 (5.99)
Ponatinib (0.10)	8.17±0.72 (0.86)	11.00±0.99 (1.16)^c^
Ponatinib (0.25)	8.33±0.69 (0.88)	6.66±0.94 (0.70)^c^
Ponatinib (0.50)	8.96±1.16 (0.94)	4.03±0.34 (0.43)^c^
Cepharanthine (2.5)	8.55±0.94 (0.90)	4.93±0.71 (0.52)^c^
Cisplatin (μM)	4354.43±358.66 (1.00)^b^	4627.31±341.80 (1.06)
Ponatinib (0.10)	4417.11±145.05 (1.01)	5395.15±159.79 (1.24)
Ponatinib (0.25)	4625.90±444.78 (1.06)	5115.46±229.46 (1.17)
Ponatinib (0.50)	4394.95±252.00 (1.01)	5286.64±319.97 (1.21)
Cepharanthine (2.5)	4489.97±379.32 (1.03)	5304.49±427.60 (1.22)

a Values represent mean ± SD of at least three independent experiments, each performed in triplicate.

b Fold of resistance was calculated as the IC_50_ values of paclitaxel, docetaxel, vincristine, vinblastine, or cisplatin of HEK/pcDNA or HEK/MRP7 cells in the absence or presence of reversal agents divided by the IC_50_ values of paclitaxel, docetaxel, vincristine, vinblastine or cisplatin of HEK/pcDNA cells without the reversing agents.

c P<0.05.

## References

[1] Goldman JM (2012). Ponatinib for chronic myeloid leukemia. N Engl J Med.

[2] Gromicho M, Dinis J, Magalhaes M (2011). Development of imatinib and dasatinib resistance: dynamics of expression of drug transporters ABCB1, ABCC1, ABCG2, MVP, and SLC22A1. Leuk Lymphoma.

[3] Breccia M, Alimena G (2008). Refining targeted therapies in chronic myeloid leukemia: development and application of nilotinib, a step beyond imatinib. Onco Targets Ther.

[4] Shah NP, Nicoll JM, Nagar B (2002). Multiple BCR-ABL kinase domain mutations confer polyclonal resistance to the tyrosine kinase inhibitor imatinib (STI571) in chronic phase and blast crisis chronic myeloid leukemia. Cancer Cell.

[5] Cortes JE, Kantarjian H, Shah NP (2012). Ponatinib in refractory Philadelphia chromosome-positive leukemias. N Engl J Med.

[6] Jones PM, George AM (2004). The ABC transporter structure and mechanism: perspectives on recent research. Cell Mol Life Sci.

[7] Davidson AL, Dassa E, Orelle C, Chen J (2008). Structure, function, and evolution of bacterial ATP-binding cassette systems. Microbiol Mol Biol Rev.

[8] Sun YL, Patel A, Kumar P, Chen ZS (2012). Role of ABC transporters in cancer chemotherapy. Chin J Cancer.

[9] Szakacs G, Varadi A, Ozvegy-Laczka C, Sarkadi B (2008). The role of ABC transporters in drug absorption, distribution, metabolism, excretion and toxicity (ADME-Tox). Drug Discov Today.

[10] Brozik A, Hegedus C, Erdei Z (2011). Tyrosine kinase inhibitors as modulators of ATP binding cassette multidrug transporters: substrates, chemosensitizers or inducers of acquired multidrug resistance?. Expert Opin Drug Metab Toxicol.

[11] Hegedus T, Orfi L, Seprodi A, Varadi A, Sarkadi B, Keri G (2002). Interaction of tyrosine kinase inhibitors with the human multidrug transporter proteins, MDR1 and MRP1. Biochim Biophys Acta.

[12] Ozvegy-Laczka C, Hegedus T, Varady G (2004). High-affinity interaction of tyrosine kinase inhibitors with the ABCG2 multidrug transporter. Mol Pharmacol.

[13] Hegedus C, Ozvegy-Laczka C, Apati A (2009). Interaction of nilotinib, dasatinib and bosutinib with ABCB1 and ABCG2: implications for altered anti-cancer effects and pharmacological properties. Br J Pharmacol.

[14] Burger H, van Tol H, Boersma AW (2004). Imatinib mesylate (STI571) is a substrate for the breast cancer resistance protein (BCRP)/ABCG2 drug pump. Blood.

[15] Brendel C, Scharenberg C, Dohse M (2007). Imatinib mesylate and nilotinib (AMN107) exhibit high-affinity interaction with ABCG2 on primitive hematopoietic stem cells. Leukemia.

[16] Shen T, Kuang YH, Ashby CR (2009). Imatinib and nilotinib reverse multidrug resistance in cancer cells by inhibiting the efflux activity of the MRP7 (ABCC10). PLoS One.

[17] Tiwari AK, Sodani K, Wang SR (2009). Nilotinib (AMN107, Tasigna) reverses multidrug resistance by inhibiting the activity of the ABCB1/Pgp and ABCG2/BCRP/MXR transporters. Biochem Pharmacol.

[18] Tiwari AK, Sodani K, Dai CL (2013). Nilotinib potentiates anticancer drug sensitivity in murine ABCB1-, ABCG2-, and ABCC10-multidrug resistance xenograft models. Cancer Lett.

[19] Dai CL, Tiwari AK, Wu CP (2008). Lapatinib (Tykerb, GW572016) reverses multidrug resistance in cancer cells by inhibiting the activity of ATP-binding cassette subfamily B member 1 and G member 2. Cancer Res.

[20] Kuang YH, Shen T, Chen X (2010). Lapatinib and erlotinib are potent reversal agents for MRP7 (ABCC10)-mediated multidrug resistance. Biochem Pharmacol.

[21] Sen R, Natarajan K, Bhullar J (2012). The novel BCR-ABL and FLT3 inhibitor ponatinib is a potent inhibitor of the MDR-associated ATP-binding cassette transporter ABCG2. Mol Cancer Ther.

[22] Malofeeva EV, Domanitskaya N, Gudima M, Hopper-Borge EA (2012). Modulation of the ATPase and transport activities of broad-acting multidrug resistance factor ABCC10 (MRP7). Cancer Res.

[23] Hopper-Borge EA, Churchill T, Paulose C (2011). Contribution of Abcc10 (Mrp7) to *in vivo* paclitaxel resistance as assessed in Abcc10^−/−^ mice. Cancer Res.

[24] Chen ZS, Hopper-Borge E, Belinsky MG, Shchaveleva I, Kotova E, Kruh GD (2003). Characterization of the transport properties of human multidrug resistance protein 7 (MRP7, ABCC10). Mol Pharmacol.

[25] Carmichael J, DeGraff WG, Gazdar AF, Minna JD, Mitchell JB (1987). Evaluation of a tetrazolium-based semiautomated colorimetric assay: assessment of chemosensitivity testing. Cancer Res.

[26] Zhou Y, Hopper-Borge E, Shen T (2009). Cepharanthine is a potent reversal agent for MRP7(ABCC10)-mediated multidrug resistance. Biochem Pharmacol.

[27] Shi Z, Liang YJ, Chen ZS (2006). Reversal of MDR1/P-glycoprotein-mediated multidrug resistance by vector-based RNA interference in vitro and in vivo. Cancer Biol Ther.

[28] van den Heuvel-Eibrink MM, van der Holt B, Burnett AK (2007). CD34-related coexpression of *MDR1* and *BCRP* indicates a clinically resistant phenotype in patients with acute myeloid leukemia (AML) of older age. Ann Hematol.

[29] Wulf GG, Wang RY, Kuehnle I (2001). A leukemic stem cell with intrinsic drug efflux capacity in acute myeloid leukemia. Blood.

[30] Lainey E, Sebert M, Thepot S (2012). Erlotinib antagonizes ABC transporters in acute myeloid leukemia. Cell Cycle.

[31] Steinbach D, Legrand O (2007). ABC transporters and drug resistance in leukemia: was P-gp nothing but the first head of the Hydra?. Leukemia.

[32] Schaich M, Soucek S, Thiede C, Ehninger G, Illmer T (2005). *MDR1* and *MRP1* gene expression are independent predictors for treatment outcome in adult acute myeloid leukaemia. Br J Haematol.

[33] List AF, Kopecky KJ, Willman CL (2001). Benefit of cyclosporine modulation of drug resistance in patients with poor-risk acute myeloid leukemia: a Southwest Oncology Group study. Blood.

[34] Coley HM (2010). Overcoming multidrug resistance in cancer: clinical studies of P-glycoprotein inhibitors. Methods Mol Biol.

[35] van der Holt B, Lowenberg B, Burnett AK (2005). The value of the MDR1 reversal agent PSC-833 in addition to daunorubicin and cytarabine in the treatment of elderly patients with previously untreated acute myeloid leukemia (AML), in relation to MDR1 status at diagnosis. Blood.

[36] Tang R, Faussat AM, Perrot JY (2008). Zosuquidar restores drug sensitivity in P-glycoprotein expressing acute myeloid leukemia (AML). BMC Cancer.

[37] Cortes JE, Pinilla-Ibarz J, le Coutre P, Paquette R, Chuah C, Nicolini FE, Apperley J, Khoury HJ A pivotal phase 2 trial of ponatinib in patients with chronic myeloid leukemia (CML) and Philadelphia chromosome-positive acute lymphoblastic leukemia (Ph+ALL) resistant or intolerant to dasatinib or nilotinib, or with the T315I BCR-ABL mutation: 12-Month Follow-up of the PACE Trial J, 2012. https://ash.confex.com/ash/2012/webprogram/Paper48561.html.

[38] Niu Q, Wang W, Li Y (2012). Low molecular weight heparin ablates lung cancer cisplatin-resistance by inducing proteasome-mediated ABCG2 protein degradation. PLoS One.

[39] Han HK, Van Anh LT (2012). Modulation of P-glycoprotein expression by honokiol, magnolol and 4-O-methylhonokiol, the bioactive components of *Magnolia officinalis*. Anticancer Res.

[40] Tang SL, Chen WJ, Yin K (2012). PAPP-A negatively regulates ABCA1, ABCG1 and SR-B1 expression by inhibiting LXRα through the IGF-I-mediated signaling pathway. Atherosclerosis.

[41] Wang H, Wang X, Li Y (2012). The proteasome inhibitor bortezomib reverses P-glycoprotein-mediated leukemia multi-drug resistance through the NF-κB pathway. Pharmazie.

[42] Wang P, Zhang Z, Gao K (2009). Expression and clinical significance of ABCC10 in the patients with non-small cell lung cancer. Zhongguo Fei Ai Za Zhi.

[43] Oguri T, Ozasa H, Uemura T (2008). MRP7/ABCC10 expression is a predictive biomarker for the resistance to paclitaxel in non-small cell lung cancer. Mol Cancer Ther.

[44] Borel F, Han R, Visser A (2012). Adenosine triphosphate-binding cassette transporter genes up-regulation in untreated hepatocellular carcinoma is mediated by cellular microRNAs. Hepatology.

